# Tetracycline Allergy

**DOI:** 10.3390/pharmacy7030104

**Published:** 2019-08-03

**Authors:** Leslie A. Hamilton, Anthony J. Guarascio

**Affiliations:** 1 Department of Clinical Pharmacy and Translational Science, the University of Tennessee Health Science Center College of Pharmacy, Knoxville, TN 37909, USA; 2 Division of Pharmacy Practice, Duquesne University School of Pharmacy, 600 Forbes Avenue, Pittsburgh, PA 15282, USA

**Keywords:** tetracycline, doxycycline, minocycline, allergy, allergic reaction

## Abstract

Despite the widespread use of tetracycline antibiotics since the late 1940s, tetracycline hypersensitivity reactions have rarely been described in the literature. A comprehensive PubMed search was performed, including allergic and serious adverse reactions attributed to the tetracyclines class of antibiotics. Of the evaluated tetracycline analogs, minocycline was attributed to the greatest overall number and severity of serious adverse events reported in the literature, with notable reactions primarily reported as respiratory and dermatologic in nature. Reactions to tetracycline have also been well described in the literature, and although dermatologic reactions are typically less severe in comparison with minocycline and doxycycline, various reports of anaphylactic reactions exist. Although doxycycline has been noted to have had the fewest reports of severe allergic reactions, rare descriptions of life-threatening reactions are still reported in the literature. Allergic reactions regarding tetracyclines are rare; however, adverse reaction type, severity, and frequency among different tetracycline analogs is somewhat variable. A consideration of hypersensitivity and adverse reaction incidence should be performed prior to the selection of individual tetracycline entities.

## 1. Introduction

Tetracyclines were first discovered in the 1940s [1] and are still widely utilized antibiotic agents with a unique spectrum of activity and clinical utility for a variety of infections. Tetracycline, doxycycline, and minocycline have been extensively studied, while doxycycline and minocycline are most frequently used due to tolerability and dosing considerations. Demeclocycline is a commercially available tetracycline rarely deployed for its antibacterial activity; rather, it is sometimes used for treatment of the syndrome of inappropriate antidiuretic hormone secretion (SIADH) [2] and therefore will not be formally reviewed. At this time, newer, enhanced spectrum tetracycline derivatives such as tigecycline and recently approved agents such as eravacycline, sarecycline, and omadacycline, have more limited data regarding their use. 

Tetracyclines display broad activity against Gram-positive aerobic bacteria and some Gram-negative bacteria, various atypical pathogens, and some protozoan parasites. A common indication for the use of doxycycline, either alone or in combination with a beta-lactam antibiotic, includes the empiric treatment of community-acquired pneumonia due to Streptococcus pneumoniae and atypical pathogen activity [3]. Doxycycline and minocycline activity against community-acquired methicillin-resistant Staphylococcus aureus (MRSA) isolates provides a valuable utility for the treatment of acute bacterial skin and skin structure infections (ABSSIs) [4,5]. 

Doxycycline and minocycline are also first-line options for the treatment of acne due to their efficacy against Propionobacterium acnes [6]. Doxycycline is an agent commonly utilized for various sexually transmitted infections such as chlamydia [7], as well as tickborne infections borelliosis (such as Lyme disease), ehrlichiosis, and rickettsial diseases (such as Rocky Mountain spotted fever) [8]. While newer tetracycline agents such as tigecycline offer an extended spectrum of activity compared with older agents, these are typically utilized as alternatives rather than first line options for resistant or polymicrobial infections.

## 2. Materials and Methods

A comprehensive literature search was completed using PubMed from 1956 to present. The search terms included tetracycline, minocycline, doxycycline, tigecycline, adverse event, allergy, sensitivity, and allergic reaction. Only articles in English were included.

## 3. Results and Discussion

The literature evaluation and discussion is divided into sections by each medication, including tetracycline, minocycline, and doxycycline. 

### 3.1. Tetracycline

Tetracycline has a long track record of the successful treatment of a variety of infections such as acne, Helicobacter pylori infection, sexually transmitted infections, and periodontitis. In current clinical practice, tetracycline use is often limited due to preferential selection of newer tetracycline derivatives such as doxycycline. Although there are various reasons for this, both administration (four times daily for tetracycline versus twice daily for doxycycline) and better tolerability with doxycycline have been well described [9]. Chlortetracycline, the first tetracycline antibiotic identified, is no longer utilized in humans, rather, it still holds utility as a veterinary antibiotic [9]. Due to its lack of current use, it was excluded from this review, despite its shared characteristics of both allergic reaction and cross-reactivity with other tetracyclines.

Most of the primary literature data regarding tetracycline reactions is described in case reports and case series studies. The most commonly reported reaction due to tetracycline administration is a fixed drug reaction. While the site of this reaction varies, including the extremities, chest, and face, and a multitude of reports describe penile lesions [10,11,12,13,14]. Fixed drug reactions have rarely been noted intraorally or on the entire body [15,16]. In addition, many patients had previously received tetracycline prior to the fixed drug incident, with one patient receiving up to five documented courses prior to the reaction [17]. Notably absent from the literature are more severe and widespread dermatologic reactions such as Stevens-Johnson syndrome (SJS) and Drug Rash with Eosinophilia and Systemic Symptoms (DRESS), which has rarely been associated with other tetracycline derivatives such as minocycline and doxycycline.

Since one of the hallmarks of a fixed drug reaction is local recurrence upon re-challenge of the offending agent, various studies chose to perform this tetracycline re-challenge when feasible and appropriate. High reactivity rates with the re-administration of tetracycline have been reported. Delaney et al. 1970 described a second dermatologic reaction to an oral challenge dose administered shortly after an initial fixed drug reaction affecting the glans penis with dysuria [14]. On various occasions, patients are prescribed subsequent courses of oral tetracycline years later for a different infection following a reaction. One such case also yielded a similar fixed drug reaction on the thigh, buttocks, and penis [18]. Yap et al. describes a case of tetracycline-induced solar urticaria that developed within five minutes of patient exposure to sunlight [19]. Follow-up photo-testing as a diagnostic challenge revealed light-induced eruptions, while these reactions were resolved within a week of tetracycline discontinuation [19]. Likewise, a case report described an atypical acneiform cutaneous eruption that occurred during primary tetracycline exposure, as well as with two different episodes of re-administration [20]. There are several others dermatologic reports that are included in Table 1 [21,22,23,24].

Overall, IgE-mediated immediate-type anaphylactic hypersensitivity reactions due to tetracycline use are rare and typically not fatal when an adequate intervention is performed (Table 2). When reported, most anaphylactic reactions noted by case reports have occurred within 10–45 min following oral or intravenous administration of tetracycline, while one case of anaphylaxis following intramuscular administration occurred rapidly in less than 20 s [25]. Since anaphylaxis can occur once a patient reacts to an antigen for which they have been previously sensitized, various cases of tetracycline-induced anaphylaxis reported prior tetracycline exposure [26,27]. 

Only one case report described re-challenging the patient’s anaphylactic reaction to tetracycline due to concomitant tracheobronchitis with the first reaction [27]. As expected, a similar anaphylactic response was noted in this individual upon re-challenge. Although skin testing following anaphylaxis from tetracycline is rarely described, one report by Ogita et al. 2011 utilized a skin prick testing method using a 250 mg tetracycline capsule dissolved in 10 mg of saline [28]. A positive test with significant erythema and a wheal greater than 5 mm in diameter was observed; however, similar prick tests performed for both minocycline and doxycycline were negative [28]. Similarly, Fellner et al., 1965, reported a positive direct skin test five days following an anaphylactic tetracycline reaction, as well as a positive hemagglutination assay test [29]. The majority of case reports did not report follow-up skin testing following anaphylaxis related to tetracycline administration [25,26,27,30,31], likely due to a lack of a standardized method for allergy confirmation as well as resources required for testing.

### 3.2. Minocycline

Much of the literature surrounding allergic and adverse reactions to the tetracycline class is gleaned from case reports and case series. As previously mentioned, minocycline is most frequently used for the treatment of acne in the outpatient setting, although it does have other uses. Minocycline is a semisynthetic tetracycline and the reactions described with it will be divided into dermatological, pulmonary, rheumatologic, and other reactions associated with its use. Minocycline received United States Food and Drug Administration (FDA) approval in 1971, so much of the literature discussing its adverse effects dates from the 1980s and 1990s. In 1975, exanthema was reported in 86 of the 22,500 patients taking minocycline by Lederle Laboratories, the original manufacturer of minocycline [32]. Minocycline has been associated with a 13.6% incidence of adverse effects, ranging from nausea and vomiting to drug-hypersensitivity syndrome [33]. Between 1998 and 2003, 72 adverse events per million prescriptions of minocycline were reported by the FDA. Unfortunately, the incidence of adverse events to minocycline are six times more common than with doxycycline [34]. Unlike the other tetracyclines, because minocycline undergoes metabolism by the liver, the adverse effects could be due to changes in the cytochrome p450 system [35].

Minocycline hypersensitivity reactions are more commonly reported in females [36]. There has also been a suggestion that the mechanism of minocycline-induced reactions is due to minocycline or a metabolite acting as superantigen, which can lead to the overactivation of lymphocytes and a considerable release of cytokines [36]. These lymphocytes can move into the skin, causing dermatologic manifestations. The development of myocarditis has a similar mechanism to dermatological involvement. Minocycline metabolites bind to myocardial collagen, causing a T-cell-mediated cascade of inflammation [34]. Eosinophils are attracted to the inflammation and cause cardiac damage through the eosinophil cationic protein, eosinophil peroxidase, and the major basic protein. Myocarditis may present as hypersensitivity, eosinophilic myocarditis, or necrotizing eosinophilic myocarditis [37]. There are some reports of viral infections triggering a decreased metabolism or the formation of reactive metabolites from minocycline causing the proliferation of T-cells and cytokine release [34]. The reactive iminoquinone metabolite may contribute to this and activate specific viruses, such as human herpesvirus type 6, which can cause a late-phase reaction [38]. Tetracycline and doxycycline are not metabolized to iminoquinone derivatives, which explains the proclivity of this reaction to be with minocycline. Reports of hypersensitivity syndrome from minocycline have described a fever that develops two to four weeks after onset of therapy, though it may take weeks or months for symptoms to resolve [39].

Drug Rash with Eosinophilia and Systemic Symptoms (DRESS) syndrome, which is associated with multiple agents, particular anticonvulsants, can also be seen with the use of minocycline. DRESS syndrome usually is associated with delayed onset and a prolonged course, with symptoms beginning between two weeks and two months after the initial medication exposure [34]. This syndrome is diagnosed by clinical criteria, which include fever, skin eruption, lymphadenopathy, fever, and facial edema, and systemic involvement can cause hepatitis, cerebral edema, and myocarditis. The risk of mortality of up to 10% with DRESS syndrome is often due to involvement with the liver and the heart [34]. The Fitzpatrick Skin Phototype Classification divides skin and eye color and characterizes them based on the skin’s reaction to sun exposure. These skin types (phototypes) are classified by the amount of melanin pigment in the skin. DRESS syndrome from minocycline is often associated with Phototypes V and VI, which includes individuals with darker skin (i.e., brown, dark brown, black) who rarely or never burn, possibly due to a melanin-minocycline complex [34,40]. Patients with these skin types might experience a more severe and prolonged course of DRESS syndrome. Previous research has also investigating the predisposition of patients of African descent who may have an altered variant of major histocompatibility complex haplotypes and an altered cytochrome p450 metabolism [34]. In a study by Maubec and colleagues, the accumulation of minocycline in patients’ skin who developed DRESS syndrome from minocycline was investigated [35]. The skin and plasma minocycline levels were obtained and genetic polymorphisms were investigated. Six of the eight patients had significant plasms levels of minocycline at 11 days and up to 17 months after the discontinuation of minocycline. Three patients had similar elevations in the skin at two to 17 months after minocycline was stopped [35]. High-performance liquid chromatography was performed and type III minocycline-related pigmentation was seen and may be due to the potential formation of a minocycline-melanin complex [35].

Additional dermatologic manifestations have been described as Sweet’s syndrome, which is an acute febrile neutrophilic dermatosis which has the characteristics of acute onset of fever, leukocytosis, and erythematous plaques with neutrophils [41]. Von den Driesch described major and minor criteria for diagnosis of Sweet’s syndrome in 1994. Major criteria include acute onset of painful erythematous plaques or nodules and neutrophilic infiltration of these plaques without leukocytoclastic vasculitis [41]. Minor criteria include a non-specific respiratory or gastrointestinal tract infection, or vaccination associated with inflammatory disease, malignancy, or pregnancy; malaise and fever; an elevated erythrocyte sedimentation rate, c-reactive protein, leukocytosis, and increased segmented-nuclear neutrophils; or an excellent response to treatment with corticosteroids or potassium iodide. At least one major and two minor criteria are required for diagnosis [41].

The adverse pulmonary manifestations seen with minocycline include lupus, hypersensitivity pneumonitis, and pleural effusions [42]. Eosinophilic pneumonia induced by minocycline has also been reported. In a case series of seven patients by Toyoshima et al., most patients presented with a dry cough, dyspnea, and fever [43]. On computed tomography (CT) scans of the chest, bilateral diffuse ground glass appearance was present in all patient, with some patients demonstrating micronodules and infiltrations. Eosinophils were also elevated in bronchoalveolar lavage specimens [43]. The authors concluded that minocycline-induced pneumonitis is manifested as eosinophilic pneumonia [43].

There have been additional reports of autoimmune syndromes caused by minocycline use. One such phenomenon has been described as a serum sickness, which is marked by fever, arthralgias, rash, and lymphadenopathy that resolve after drug discontinuation [44,45,46,47]. Serum sickness-like reactions often present within six to 21 days after administration of the causative agent [48]. Like other reports of reactions from minocycline, incidence in women appears to be greater than men. In two cases, the patients had previously tolerated tetracycline without incident [45]. In addition, one of these patients developed angioedema, which required epinephrine and also became worse when taking nonsteroidal anti-inflammatory agents [45]. Reports of lupus-like syndromes have also been reported, which are distinguished by characteristics of systemic lupus erythematosus (SLE), in addition to positive anti-nuclear antibodies (ANA) [44,46,48]. In the reported cases, in addition to most of the patients being female, most were young women being treated for acne with minocycline who had arthralgias and had an elevated erythrocyte sedimentation rate and positive ANA. In the reported cases of minocycline-induced hepatitis, the incidence appears to be higher in males than females [44]. In addition to rash, fever, and arthralgias, transaminitis was present in all patients, along with a positive ANA in most patients. While hepatitis resolved after discontinuation of the causative agent, the resolution of liver enzymes was a much slower process when compared to the resolution of symptoms in minocycline-induced lupus [44].

Vasculitis has also been reported with similar symptoms as other autoimmune conditions, in addition to the manifestation of livedo reticularis. Perinuclear antineutrophil cytoplasmic antibodies (pANCA) were present with a high titer in patients with minocycline-induced vasculitis [44]. In a report by Lan et al., autoimmune manifestations developed well after the initial insult [49]. Their case describes a 13-year-old girl who developed type 1 diabetes mellitus five months after the initial presentation, in addition to rapid-onset alopecia [49]. It has been suggested that viral reactivation may also play a role in the development of autoimmune conditions after DRESS syndrome. A report by Brown et al. described a 15-year-old female who presented with fever, erythroderma, lymphadenopathy, and facial swelling after a four-week course of minocycline [50]. Several months later, the patient was diagnosed with Graves disease, which required radioactive iodine thyroid ablation and type 1 diabetes mellitus. Another report showed the development of polyarteritis nodosa in a 23-year-old female [51]. The patient developed subcutaneous nodules, livedo reticularis, and pigmented lesions on her lower limbs and pANCA was positive. Finally, Rahman et al. described a case of rhabdomyolysis from minocycline in a 20-year-old female who was a professional ballet dancer [52]. In addition to erythematous eruptions, arthralgias, and facial swelling, she also developed significant rhabdomyolysis, which resolved with prednisone therapy.

There are a few reports of neurologic and psychiatric manifestations from minocycline [53,54,55,56,57]. An uncommon effect of minocycline was reported in one case where the patient developed depersonalization symptoms [53]. Depersonalization is characterized by a decrease in self-awareness, patients reporting disembodiment and emotional numbness. In this report, the patient felt disconnected and lost, with a feeling like she was disconnected from her body [53]. Upon discontinuation of minocycline, the symptoms resolved within two to three days. A report by Lefebvre and colleagues describes a patient with meningitis and cerebral edema induced by minocycline [54]. A 31-year-old female with human immunodeficiency virus (HIV) developed headaches, facial edema, and rash after taking minocycline, presenting with eosinophilia, leukocytosis, cytolytic hepatitis, and elevated c-reactive protein. A lumbar puncture showed lymphocytic meningitis and a CT scan of the head demonstrated diffuse cerebral edema [54]. Minocycline was discontinued and treatment was initiated with prednisolone 60 mg orally daily and the patient fully recovered after a slow taper of the prednisolone. This case suggests a potential risk of such a syndrome in the HIV population. A 35-year-old female developed non-enhancing foci with restricted diffusion on a brain magnetic resonance image (MRI) in the parietal and occipital lobes [55]. In addition to her neurological manifestations, she also demonstrated pulmonary involvement. The patient received corticosteroids and her neurologic abnormalities almost completed resolved by six weeks [55]. 

Another report describes anaphylaxis from minocycline in a 56-year-old female [56]. The patient presented with urticarial, angioedema, dyspnea, and hypotension on three separate occasions and was treated for anaphylaxis with chlorpheniramine, famotidine, hydrocortisone, and epinephrine. After the discovery of the causative agent, the patient fully recovered and has experience no further anaphylaxis [56]. Okano et al. 1995 described anaphylaxis in a middle-aged female within 30 min of receiving 100 mg of oral minocycline [57]. A minocycline scratch skin test was performed which revealed a large (13 × 16 mm) wheal and significant surrounding erythema [57]. This skin test helped to confirm minocycline allergy due to concomitant medication skin test and oral challenge tests, revealing a negative result. Other reports of adverse reactions to minocycline are listed in Table 3 and Table 4 [58,59,60,61,62,63,64,65,66,67,68,69,70]

### 3.3. Doxycycline

Doxycycline is typically recognized as the most tolerable agent within the tetracycline class. Likewise, it is typically utilized to a greater degree compared with other tetracycline counterparts. The fairly limited body of literature describing doxycycline hypersensitivity and associated side effects reflects the overall tolerability of this agent. One such report of generalized urticaria following doxycycline was found to be dependent on the drug formulation itself, raising the possibility that an impurity or contaminant during doxycycline synthesis was to blame rather than the parent compound [71].

Few case reports have described serious reactions following doxycycline administration. A type I, immediate type anaphylactic reaction was described in a 71-year-old woman who received intravenous doxycycline in combination with beta-blocker administration during general anesthesia [72]. The patient experienced acute bronchospasm, hypotension, and urticaria, and required both intubation and medical support, which included epinephrine. While there is a lack of additional published reports regarding anaphylaxis, rare reports to the manufacturer (post-marketing surveillance) have described similar symptoms, such as bronchospasm and hypotension, that required medical intervention, including at least one fatality [72]. 

Most reports of adverse reactions following doxycycline therapy involve dermatologic reactions. Although more commonly reported with other tetracycline derivatives, skin eruptions have been reported with doxycycline use [73,74]. Of note is one case that described cutaneous eruptions after two full years of doxycycline therapy [73]. Erythematous plaques and nodules were noted on the patient’s extremities, and the patient’s sensitivity to this reaction was following an additional drug challenge a year later. Another case describes a 49-year-old male with recurrent, erythematous and purple patches on his hands and penis following intermittent oral doxycycline 100 mg twice daily for four months [74]. Once again, a 100 mg challenge dose confirmed this reaction when his patches returned within an hour. 

Various reports, albeit rare, describe Stevens-Johnson syndrome (SJS) following doxycycline administration [75,76,77,78]. Although SJS typically results in epidermal detachment in less than 10% of the body surface area, morbidity and mortality can be significant. In addition to widespread cutaneous eruptions, typical presentation described with doxycycline-induced SJS typically also include oral mucosa lesions [75,76,77] and/or ophthalmic lesions [76,77]. Notably, reports of doxycycline-induced SJS were found to develop both immediately following doxycycline administration (within 24 h) [76] as well as within approximately 10–15 days [75,77] using standard doses of 200 mg per day or, in one case, higher doses of 300 mg daily [77]. Despite prompt intervention, long-term ophthalmologic deficits from ocular involvement have been reported [76]. 

Other severe reactions have been infrequently reported with doxycycline use, including subacute cutaneous lupus erythematosus in an 84-year-old female despite sun avoidance [79]. Diffuse scaly papules and plaques were observed on her chest, abdomen, and extremities, while laboratory and pathology data suggested presumptive diagnosis of lupus erythematosus. DRESS syndrome has also been reported following three weeks of doxycycline treatment in an otherwise healthy 20-year-old female with acne [80]. Her initial presentation included papular eruptions on her face that later manifested into a widespread popular rash on her face, trunk, and extremities. She developed tonsillar edema with eosinophilia along with pulmonary edema and pneumonitis requiring intubation. Histological findings helped confirm DRESS syndrome diagnosis.

Shapiro et al. made recommendations as to who should avoid tetracycline antibiotics [81]. Minocycline should not be used in patients with a history of SLE or with a history of SLE in a first-degree relative. Caution should also be exercised with patients who have underlying hepatic or renal disease [81]. In addition, patients who develop a hypersensitivity reaction, serum sickness, or drug-induced lupus to any of the tetracyclines should avoid the class altogether. While many of the reactions to the tetracycline occur within the first few months of therapy, late reactions can occur years after the initiation of therapy [81].

## 4. Tetracycline Cross-Reactivity

Various studies have noted patients with a penicillin allergy also exhibiting an allergic response to tetracycline [26,29,30]. Some researchers have wonder if a penicillin allergy may predict tetracycline sensitivity. If such a relationship between penicillin and tetracycline allergy were to exist, this would be clinically significant, since tetracyclines are often chosen as an alternative therapy for infections in patients with penicillin allergy. However, an immunologic study of one such patient that utilized direct skin testing, passive transfer analysis, and hemagglutination testing determined two different and unrelated antibody-mediated responses for penicillin and tetracycline sensitivities, respectively [29]. These data contradict the presence of a direct relationship between penicillin and tetracycline allergy.

Few studies have directly evaluated tetracycline and minocycline testing for cross-sensitivity between the agents. Chan et al. completed two studies evaluating this relationship [82]. In the first study, six patients with a known fixed drug eruption from tetracycline were re-challenged with increasing doses of tetracycline until a reaction was seen. In the next study, the same patients were given either minocycline or chlortetracycline [82]. One of the four patients given minocycline had a reaction and one of the two patients given chlortetracycline reacted. The authors hypothesized, based on a limited data set, that one in four patients with a fixed drug eruption with tetracycline would react to another agent in the class [82]. Another study described similar cross-reactivity between doxycycline and minocycline. A patient with a prior history of fixed drug reaction due to doxycycline had a recurrence of this reaction when minocycline was administered eight months following the initial reaction. Each of these reactions occurred within hours of administration [83]. Other studies did not report the same incidence of cross-reactivity between tetracycline, minocycline, and doxycycline. One such report describes two cases of tetracycline-induced balanitis that did not recur with subsequent doxycycline or minocycline oral challenges [84]. Likewise, these limited reports indicate that tetracycline cross-reactivity due to dermatologic manifestations can be variable.

## 5. Conclusions

Compared to other classes of antibiotics, the tetracyclines collectively have lower rates of immediate-type IgE-mediated hypersensitivity reactions, although various other serious allergic reactions have been described. Primary literature reports of type I allergic reactions due to tetracycline derivatives are limited to case reports. Minocycline carries the greatest risk of serious, non-IgE mediated reactions that are often dermatologic and pulmonary in nature, although many reports were in patients who received prolonged courses of therapy. Tetracycline has been associated with less severe dermatologic reactions, such as fixed drug reaction, while doxycycline may carry the best overall safety profile regarding allergic reaction potential. Data describing allergic cross-reactivity within the tetracycline class is primarily limited to describing the development of fixed drug reactions.

## Figures and Tables

**Table 1:** Dermatological adverse effects caused by tetracycline.

Publication	Patient(s)	Manifestations	Treatment and Outcome
Ziprowski 1958	35 yo female	Erythematous rash on face, upper extremities, and entire body including mouth and tongue mucous membranes, joint pain	Antihistamine, survived
Calnan 1960	52 yo male	Vesicular eruptions with erythema, edema, and papules and pustules of hands and feet	Calamine lotion, phenobarbitone, diphenhydramine, survived
Dubowitz 1964	5 yo male	Urticarial rash followed by swelling and pigmentation of face, eyelids, and extremities	Not reported, survived
Minkin 1969	38 yo male	Erythema, pruritic and pigmented patches, fixed drug reaction of finger webs of hand	Not reported, survived
Broden 1970	Male, age unknown	Erythema, pruritic patch on index finger and penis, fixed drug reaction	Not reported, survived
Delaney 1970	49 yo male	Erythema, pruritic rash on penis	Steroid cream, antihistamine, survived
Tarnowski 1970	44 yo female	Erythematous, pigmented slightly raised plaques on extremities	Not reported, survived
Bean 1971	30 yo male	Acneiform eruption, superficial follicular pustules on neck, chest, extremities	Prednisone, calamine lotion, survived
Csonka 1971	24 yo male; 29 yo male; 34 yo male; 21 yo male	All: erythematous fixed drug eruption on penis	Not reported, survived
Armati 1973	36 yo male	Erythema, pruritic macules on penis, buttocks and thighs	Hydrocortisone cream, survived
Parish 1978	29 yo female	Erythema, edematous plaque, pulsating fixed drug reaction of arm	Betamethasone gel, survived
Fiumara 1981	29 yo male; 22 yo male	Pigmented penile rash, fixed drug eruption (both)	Not reported, survived
Murray 1982	46 yo male	Erythematous reaction on left palate, intraoral fixed drug reaction	Not reported, survived
Dodds 1985	77 yo male; 38 yo male 56 yo male	All: balanitis, erythema, swelling, fixed drug reaction of glans penis; one with hyperpigmented lesions; one with ulcerations and hemorrhagic rash	Not reported, survived
Yap 2000	28 yo female	Solar urticaria with papular eruption of perioral and eyelid regions, reaction prominent beyond borders of clothing	Not reported, survived

yo = year old.

**Table 2:** Anaphylactic adverse effects caused by tetracycline.

Publication	Patient(s)	Manifestations	Treatment and Outcome
Sakamoto 1956	43 yo male	Loss of consciousness, convulsion, hypotension	Epinephrine, mephentermine, survived
Haas 1957	48 yo male	Wheezing, hives, angioedema, hypotension	Ephedrine, diphenhydramine, aminophylline, survived
Fellner 1965	44 yo male	Dyspnea, dizziness, flushing, tachycardia, urticaria	Epinephrine, survived
Barnett Jr. 1967	28 yo male	Wheezing, urticaria, neck pruritis	Epinephrine, diphenhydramine, survived
Singh 1977	25 yo male	Widespread urticarial rash, cyanosis, hypotension, cardiac arrest	Not reported, expired
Steinbruegge 1980	26 yo male	Generalized urticaria, angioedema	Epinephrine, diphenhydramine, survived
Ogita 2011	17 yo female	Hives, dyspnea, generalized urticaria	Chlorpheniramine, hydrocortisone, prednisolone

yo = year old.

**Table 3:** Dermatological adverse effects caused by minocycline.

Publication	Patient(s)	Manifestations	Treatment and Outcome
Shelley 1973	65 yo male	Erythematous eruption, maculopapular rash, symptoms reappeared on re-challenge	Not reported
Shimizu 1977	26 yo male	Erythematous rash, eosinophilia, symptoms reappeared on re-challenge	Corticosteroid
LePaw 1983	48 yo male	Erythematous and scaly eruption, symptoms reappeared on re-challenge	Topical steroids, survived
Shoji 1987	36 yo male	Stevens-Johnson Syndrome (fever, erythema multiforme-like eruptions, involvement of mouth and conjunctiva)	Oral corticosteroid, survived
Davies 1989	16 yo male	Erythematous and exfoliative rash, fever, lymphadenopathy, leukopenia, transaminitis, positive streptococcal antibody	Not reported, survived
	17 yo female	Macular, exfoliative rash, fever, leukopenia, transaminitis, positive streptococcal antibody	Liver transplant, expired
Kaufmann 1994	35 yo female	Rash, arthralgias, fever, lymphadenopathy, severe neutropenia	Prednisone, survived
Parneix-Spake 1995	15 yo male	Fever, diffuse pustular eruption, lymphadenopathy, leukocytosis, eosinophilia, transaminitis	Prednisone, expired (myocardial necrosis with interstitial eosinophilic infiltrate)
	17 yo female	Fever, nonfollicular pustules, lymphadenopathy, dyspnea, leukocytosis, eosinophilia, transaminitis	Prednisone, survived
Knowles 1996	6 patients (4 female/2 male)	Rash, hepatic involvement, lymphadenopathy, one patient developed Stevens-Johnson Syndrome (required liver transplant)	5/6 patients received corticosteroids, all survived
Okano 1996	27 yo female	Erythematous eruption, dyspnea	Not reported
MacNeil 1997	17 yo female	Leukocytosis, eosinophilia, fever, transaminitis, macular eruption, lymphadenopathy, vesicular rash	IV methylprednisolone, survived
Colvin 2001	13 yo male	Erythematous eruption, shock, leukocytosis, eosinophilia, transaminitis, lymphadenopathy	Antibiotics, methylprednisolone, survived
Tsuruta 2006	20 yo female	Fever, lymphadenopathy, eosinophilia, lymphocytosis, rash, transaminitis, symptoms reappeared on re-challenge	Prednisolone, survived
Shaughnessy 2010	38 yo female	Erythematous eruption and desquamation, fever, tachycardia, hypotension, increased CRP, eosinophilia, transaminitis, leukocytosis, myocarditis	IV dexamethasone, IV methylprednisolone, plasmapheresis, rituximab, survived
Kalai 2012	49 yo female	Erythematous nodules and plaques, transaminitis	Prednisolone, Sweet Syndrome, survived
Travassos 2012	19 yo female	Subcutaneous erythematous nodules and pustules, fever, increased CRP and ESR, history of Hansen’s disease, erythema nodosum leprosum	Prednisolone and treatment for multi-bacillary leprosy, survived
Kanno 2014	60 yo female	Erythematous rash, fever, periorbital edema, lymphadenopathy, leukocytosis, eosinophilia, increased CRP and ESR	IV immunoglobulin, methylprednisolone, mechanical ventilation, percutaneous cardiopulmonary support and intra-aortic balloon pump, survived
Wu 2014	46 yo female	Fever, arthralgia, transaminitis, lymphadenopathy, erythematous rash, eosinophilia, interstitial nephritis, myocarditis	Methylprednisolone, expired (eosinophilic and giant cell myocarditis)
Lan 2016	13 yo female	Eosinophilia, leukocytosis, fever, transaminitis	Required liver transplantation, survived
Gowani 2018	18 yo female	Macular rash, lymphadenopathy, fever, transaminitis, leukocytosis	Prednisone, survived

C-reactive protein: CRP; ESR: erythrocyte sedimentation rate; IV: intravenous; Year old: yo.

**Table 4:** Pulmonary adverse effects caused by minocycline.

Publication	Patient(s)	Manifestations	Treatment and Outcome
Toyoshima 1996	7 patients (4 female/3 male)	Leukocytosis, elevated erythrocyte sedimentation rate and CRP, eosinophilia, ground glass opacities on computed tomography of chest	3 patients required corticosteroids, all survived
Christodoulou 1999	16 yo female	Dyspnea, hypoxia, pulmonary infiltrates, transaminitis	Corticosteroids
Clayton 199	26 yo female	Respiratory distress, erythematous eruption tonic-clonic seizures, transaminitis, eosinophilia	Mechanical ventilation, corticosteroids, survived
Parc 2002	28 yo female	Severe asthma exacerbation, conjunctival infiltrates, eosinophilia	Prednisone, survived
Oddo 2003	54 yo female	Tachycardia, tachypnea, hypotension, fever, leukocytosis, increased CRP, eosinophilia	Mechanical ventilation, IV methylprednisolone, survived
Roca 2003	22 yo female	Acute respiratory distress syndrome, eosinophilia, rash, leukocytosis, bilateral diffuse infiltrate on chest radiograph	Mechanical ventilation, corticosteroids, antibiotics

C-reactive protein: CRP; IV: intravenous; Year old: yo.
